# Detection of *Plasmodium falciparum* DNA in saliva samples stored at room temperature: potential for a non-invasive saliva-based diagnostic test for malaria

**DOI:** 10.1186/s12936-017-2084-5

**Published:** 2017-10-27

**Authors:** Kenji O. Mfuh, Samuel Tassi Yunga, Livo F. Esemu, Obase Ngemani Bekindaka, Jessica Yonga, Jean Claude Djontu, Calixt D. Mbakop, Diane W. Taylor, Vivek R. Nerurkar, Rose G. F. Leke

**Affiliations:** 1 Department of Tropical Medicine, Medical Microbiology and Pharmacology, John A. Burns School of Medicine, University of Hawaii at Manoa, 651 Ilalo Street, Honolulu, HI 96813 USA; 2 Biotechnology Center, University of Yaoundé I, Yaoundé, Cameroon; 3 National Medical Research Institute, Yaoundé, Cameroon

**Keywords:** *Plasmodium falciparum*, Malaria, Diagnosis, Saliva, PCR, DNA

## Abstract

**Background:**

Current malaria diagnostic methods require blood collection, that may be associated with pain and the risk of transmitting blood-borne pathogens, and often create poor compliance when repeated sampling is needed. On the other hand, the collection of saliva is minimally invasive; but saliva has not been widely used for the diagnosis of malaria. The aim of this study was to evaluate the diagnostic performance of saliva collected and stored at room temperature using the OMNIgene^®^•ORAL kit for diagnosing *Plasmodium falciparum* malaria.

**Methods:**

Paired blood and saliva samples were collected from 222 febrile patients in Cameroon. Saliva samples were collected using the OMNIgene^®^•ORAL (OM-501) kit and stored at room temperature for up to 13 months. Thick blood film microscopy (TFM) was used to detect *P. falciparum* blood-stage parasites in blood. Detection of *P. falciparum* DNA in blood and saliva was based on amplification of the multi-copy 18 s rRNA gene using the nested-polymerase chain reaction (nPCR).

**Results:**

Prevalence of malaria detected by TFM, nPCR-saliva and nPCR-blood was 22, 29, and 35%, respectively. Using TFM as the gold standard, the sensitivity of nPCR-saliva and nPCR-blood in detecting *P. falciparum* was 95 and 100%, respectively; with corresponding specificities of 93 and 87%. When nPCR-blood was used as gold standard, the sensitivity of nPCR-saliva and microscopy was 82 and 68%, respectively; whereas, the specificity was 99 and 100%, respectively. Nested PCR-saliva had a very good agreement with both TFM (kappa value 0.8) and blood PCR (kappa value 0.8). At parasitaemia > 10,000 parasites/µl of blood, the sensitivity of nPCR-saliva was 100%. Nested PCR-saliva detected 16 sub-microscopic malaria infections. One year after sample collection, *P. falciparum* DNA was detected in 80% of saliva samples stored at room temperature.

**Conclusions:**

Saliva can potentially be used as an alternative non-invasive sample for the diagnosis of malaria and the OMNIgene^®^•ORAL kit is effective at transporting and preserving malaria parasite DNA in saliva at room temperature. The technology described in this study for diagnosis of malaria in resource-limited countries adds on to the armamentarium needed for elimination of malaria.

## Background

*Plasmodium falciparum* malaria remains one of the most important infectious diseases in sub-Saharan Africa (SSA). Even with the significant reduction in malaria morbidity and mortality since the beginning of the third millennium, 214 million people (88% living in SSA) acquired malaria and 438,000 people (90% from SSA) died from malaria in 2015 [1]. Transmission-blocking interventions, such as insecticide-treated nets, have been hailed as significant contributors to the decrease in the number of malaria cases and deaths. The proportion of children less than 5 years old in SSA who sleep under insecticide-treated nets increased from < 2% in 2000 to about 68% in 2015 [1]. However, such gains are threatened by the emergence of drug-resistant parasites and mosquitoes resistant to insecticides. Early diagnosis and prompt treatment of cases are of extreme importance. The ideal diagnostic method that would be most beneficial for eliminating malaria needs to be rapid, simple to perform, inexpensive, sensitive, accurate and non-invasive.

Currently available diagnostic methods for malaria include the identification of malaria parasites or parasite proteins in blood by microscopy and rapid diagnostic tests (RDT) and parasite DNA by polymerase chain reaction (PCR). Microscopic examination of Giemsa-stained thick blood film is the diagnostic gold standard, but even trained microscopists routinely detect only 50–100 parasites or more/microlitre of peripheral blood [2, 3], thus missing infected individuals with very low parasitaemia [4, 5]. People living in low malaria transmission areas harbour submicroscopic parasitaemia [6, 7] and constitute reservoirs that sustain transmission [8]. RDTs detect malaria parasite-specific proteins, such as histidine-rich protein-2 (HRP-2) in plasma. RDTs are easy to perform and suitable for field settings. Although multi-survey analyses show a strong association between prevalence of malaria by RDT and by microscopy [9], RDTs detect more infections than microscopic examination [2, 4, 10]. On the other hand, HRP-2-based RDTs may show false-positive results for up to a month following parasite clearance [5, 10–12]. Molecular techniques are highly sensitive with PCR detecting as low as 1–5 parasites/µl of blood [13–16]. Despite differences in their procedures and performances, microscopy, RDT, and nested PCR (nPCR) for malaria diagnosis share a common problem, that is, the requirement of blood samples. Collection of blood is not risk-free. Pain is experienced and phobias linked with finger pricks and needles have been clinically described [17–19]. Also, cultural and religious myths discourage some individuals from providing blood samples. These factors reduce participant compliance in epidemiological surveys where repeated sampling is required. Furthermore, accidental transmission of blood-borne pathogens such as HIV and hepatitis viruses can occur during blood draws and finger-pricks. A better method for malaria diagnosis is needed that incorporates the accuracy of PCR and the use of non-invasive clinical specimens.

Human saliva is readily available and is increasingly being recognized as an important diagnostic specimen. While more than 98% of human saliva is composed of water [20], the salivary fluid contains many constitutive and infiltrating electrolytes, proteins, and DNA that have been explored for diagnoses and monitoring of a broad variety of diseases [21–23]. For example, molecular markers of malignancy, such as p53 mutations and carcinoembryonic antigen have been detected in saliva of patients with oral carcinomas [24], and saliva concentrations of some biomarkers of cardiovascular disease correlate with corresponding serum concentrations [25]. Also, saliva-based kits have been developed for the diagnosis of HIV [26] and human papilloma virus [27].

In the field of malaria diagnostics, there have been reports of the detection of malaria parasite HRP-2, lactate dehydrogenase and *P. falciparum* DNA in the saliva of infected individuals [28–34]. Although some investigators have also detected malaria parasite DNA in urine samples, the sensitivity of saliva-based detection is significantly higher than urine-based detection of malaria parasite DNA [30–34]. Thus, saliva is a more promising non-blood alternative for malaria diagnosis than urine. The performance of PCR using saliva compared to blood for detecting *Plasmodium* DNA varies due to differences in experimental and analytic methods used by different laboratories [29–31, 33, 34].

Storage condition of saliva samples from the point of collection can affect DNA stability and PCR sensitivity [30]. In most studies that have investigated saliva as a diagnostic medium for malaria parasites, saliva samples were refrigerated or frozen from the point of collection until DNA extraction [30–34]. While this approach is valid, it is costly to maintain and not feasible in many resource-limited and remote areas. Presently, no study has evaluated the performance of saliva samples collected and stored at room temperature for the diagnosis of malaria. Progress in basic science research has led to the development of kits that can be used to collect and preserve microbial nucleic acid, including the OMNIgene^®^•ORAL saliva collection and microbial DNA stabilization kit designed to maintain the stability of total DNA in saliva at room temperature [35]. The present study, was aimed at evaluating the performance of saliva-based nested PCR for detecting *P. falciparum* DNA in saliva collected in the OMNIgene^®^•ORAL (OM-501) kit and stored at room temperature for up to 13 months, from suspected malaria patients in Cameroon.

## Methods

### Study design and population

A cross-sectional study was conducted from October 2014 to April 2015 in selected health care facilities in three regions of Cameroon, i.e., the Far North, Centre, and Northwest regions. In the Far North region, the study was conducted in the Maroua regional hospital; the Catholic Health Center Nkolbisson was the study site in the Center region; while the PMI Nkwen Bamenda and the Mother of Mary Hospital Widikum were study sites in the Northwest region. Malaria transmission is perennial in both the Center and North West regions, but peak transmission occurs at different times of the year; whereas, malaria transmission is seasonal in the Far North region. All three regions were selected as part of an on-going study aimed at characterizing the etiologies of non-malarial fevers in Cameroon. Individuals at least 2 years old with axillary temperature above 37.5 °C at presentation or complaint of fever within 24 h preceding enrollment were included in the study.

Informed consent was obtained from eligible participants who were above 18 years. Parents or legal guardians of younger children gave written informed assent on behalf of their children. Ethical approvals were obtained from the Committee on Human Subjects of the University of Hawaii (protocol number CHS 21724) and the National Research Ethics Committee of the Ministry of Public Health Cameroon (protocol Number 2014/04/442/CE/CNERSH/SP). Administrative approvals were also obtained from the directors of the various health institutions and from the Ministry of Public Health, Cameroon.

### Sample collection and storage

After informed consent had been obtained, paired blood and saliva samples were collected from each participant. Approximately 2 ml of venous blood was drawn into an EDTA tube. Blood samples were transported in cold boxes to the laboratory where they were aliquoted and stored at − 20 °C until DNA extraction. Participants were requested to dispense approximately 1 ml of saliva into OMNIgene^®^•ORAL (OM-501) kit (DNA Genotek, Ottawa, Ontario, Canada) following recommendations from the manufacturer. To avoid blood contamination of saliva, individuals with overt gum bleeding or who complained of pain in any part of their mouth were excluded. All saliva samples were stored at room temperature from the time of collection until DNA extraction (about 2–6 weeks). After 12–13 months of storage at room temperature DNA was re-extracted from saliva aliquots of patients who original tested positive for *P. falciparum* DNA in saliva.

### Malaria microscopy

Thick blood smears were prepared, stained with 10% Giemsa and read by two trained microscopists. A slide was considered positive if one or more blood-stage malaria parasites was detected after reading at least 100 high power fields. Parasitaemia (parasites/µl of blood) was determined by counting the number of parasites against 200 white blood cells and multiplying a constant of 8000 white blood cells/µl of blood. Each slide was read by two experienced microscopists and their parasitaemia readings were averaged. Discrepancies greater than 10% between two readings were resolved by the reading of a third technician.

### DNA extraction and nested PCR

DNA was extracted from 200 µl whole blood and 200 µl of saliva using NucleoSpin columns (Macherey–Nagel, Duren Germany), following manufacturer’s instructions. Purified DNA from each sample was eluted into 100 µl of elution buffer and stored at − 20 °C until nested PCR was conducted, usually within 48 h. The gene target for PCR amplification was the multicopy 18 s rRNA plasmodial gene using standard protocols [35, 36]. For each sample, a 20 µl PCR reaction mix was prepared consisting of 10 µl of 2 × GoTaq^®^Green Master Mix (Promega, Madison USA), 6 µl of nuclease-free water, 2 µl of template DNA and 1 µl each of 5 µM forward and reverse primers. For the first step of the nested PCR, eluted DNA template and genus-specific forward and reverse primers were used (rPLU5: 5′-CCTGTTGTTGCCTTAAACTTC-3′ and rPLU6: 5′-TTAAAATTGTTGCAGTTAAAACG-3′). For second step of the nested PCR, first step nested PCR amplification product was used as template along with *P. falciparum*-specific primers (rFAL1: 5′-TTAAACTGGTTTGGGAAAACCAAATATATT-3′and rFAL2: 5′-ACACAATGAACTCAATCATGACTACCCGTC-3′) [36, 37]. Purified *P. falciparum* DNA, extracted DNA from a malaria-naive individuals and molecular grade water were used as positive, negative and no template controls, respectively. Reaction conditions used have been described elsewhere [36, 37]. Nest-2 products were electrophoresed on ethidium bromide-stained 2% agarose gels and visualized on the Gel Doc™ XR+ System (Bio-Rad, USA). Samples were qualitatively scored as positive or negative if 205-bp bands were present or absent.

### Statistical analysis

Descriptive analyses were performed with statistical analysis programs for Apple Mac computer (AnalystSoft Inc. version 5) and Graph Pad Prism (version 6). Sensitivity, specificity, positive predictive value (PPV), and negative predictive value (NPV) were used as standard parameters to assess diagnostic performance. The κ coefficient was used to estimate agreement on a scale of 0–1, after correcting for agreement due to chance, between saliva nPCR and microscopy or blood nPCR results. Diagnostic test performance was conducted on MedCalc software (version 16.8). P values < 0.05 were considered significant. Paired t test was used to compare the differences between DNA concentration in saliva samples when they were initially collected and 12–13 months later. The Mann–Whitney non-parametric test was used to compare initial peripheral parasitaemia of saliva samples that remained positive after 12–13 months at room temperature and saliva samples that were initially positive but became negative after 12–13 months of storage.

## Results

**Fig. 1:**
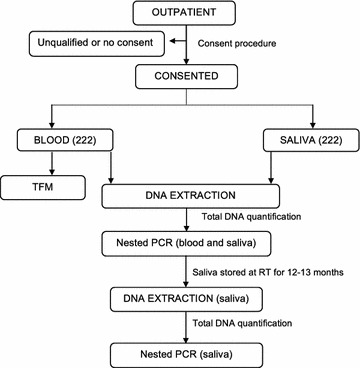
Study design for enrollment, sample collection and laboratory assays. *TFM* thick film microscopy; *RT* room temperature

Matched blood and saliva samples were collected from 222 participants living in three different geographical regions of Cameroon (Fig. 1). More females (n = 158 [71%]) than males (n = 64 [29%]) were enrolled in this study. The mean age of the study participants was 22 years (range 2 to 76 years) with age distribution as follows: 2–10 years (n = 60 [27%]), 11–20 years (n = 47 [21%]), 21–30 years (n = 93 [42%]) and ≥ 40 years (n = 22 [10%]). The prevalence of malaria by TFM, nPCR-blood, and nPCR-saliva was 24, 35, and 28%, respectively (Table 1). There were 25 (32%) malaria infections detected by nPCR-blood that were slide negative (i.e., submicroscopic infections), while nPCR-saliva identified 13 (20%) more malaria infections than TFM. The overall agreement of microscopy results between the two technicians was 98%.

**Table 1:** Baseline *P. falciparum* prevalence by different test methods

Test method	Number of samples		Prevalence (%)
	Positive	Negative	
Thick film microscopy	53	169	24
nPCR-blood	78	144	35
nPCR-saliva	66	156	28

To determine the diagnostic test performance of nPCR-saliva and nPCR-blood, TFM was used as the gold standard. All samples that were malaria-positive by microscopy were also identified by nPCR-blood giving a sensitivity of 100%, while nPCR-saliva correctly identified 95% of microscopy-positive samples giving a sensitivity of 95%. However, the specificity of nPCR-saliva and nPCR-blood were 93% and 87% compared to microscopy, respectively. nPCR-saliva and nPCR-blood had a “very good” (kappa 0.8) and “good” agreement (kappa 0.7), respectively, with TFM for the diagnosis of malaria (Table 2).

**Table 2:** Diagnostic test performance of nPCR-saliva and nPCR-blood with thick film microscopy as reference standard in the diagnosis of malaria

Test characteristics	NPCR-saliva	NPCR-blood
Sensitivity [95%CI]	95% [85 to 99]	100% [94 to 100]
Specificity [95%CI]	93% [88 to 99]	87% [80 to 91]
PPV [95%CI]	83% [72 to 91]	72% [61 to 82]
NPP [95%CI]	98% [94 to 99]	100% [97 to 100]
PLR [95%CI]	14 [8 to 25]	7 [5 to 11]
NLR [95%CI]	0.06 [0.02 to 0.17]	0
Observed agreements	94%	90%
Kappa value	0.8	0.7

*PPV* positive predictive values, *NPP* negative predictive values, *PLR* positive likelihood ratio, *NLR*, negative likelihood ratio

When nPCR-blood was used as the reference standard, the sensitivity of nPCR-saliva was 82%. Moreover, nPCR-saliva had a “very good” agreement (kappa 0.8) with nPCR-blood for diagnosis of malaria (Table 3).

**Table 3:** Diagnostic test performance of nPCR-saliva and thick film microscopy with nPCR-blood as reference standard in the diagnosis of malaria

Test characteristic	NPCR-saliva	Thick film microscopy
Sensitivity [95%CI]	82% [72 to 90]	68% [56 to 78]
Specificity [95%CI]	99% [95 to 100]	100% [97 to 100]
PPV [95%CI]	97% [89 to 99]	100% [93 to 100]
NPV [95%CI]	91% [85 to 95]	85% [79 to 90]
PLR [95%CI]	59% [15 to 235]	
NLR [95%CI]	0.2 [0.11 to 0.29]	0.32 [0.23 to 0.44]
Observed agreements	93%	89%
Kappa value	0.8	0.7

PPV, positive predictive values; NPP, negative predictive values; PLR, positive likelihood ratio; NLR, negative likelihood ratio

To determine the performance of nPCR-saliva with respect to parasitaemia and age, malaria slide-positive data were stratified into different parasitaemia and age groups and the sensitivity and specificity calculated for each group. There were three false-negative samples with parasitaemia ranging from < 100-(one sample) and 1000–2000-parasites/µl of blood (two samples). At parasitaemia > 10,000 parasites/µl of blood, the sensitivity and specificity of nPCR-saliva were 100% each. Meanwhile, the sensitivity of nPCR-saliva was 100% in the age group > 40 years (Tables 4 and 5).

**Table 4:** Sensitivity of nPCR-saliva with respect to parasitaemia in the diagnosis of malaria

Parasitaemia/µl	n	Test characteristics			
		TP	FN	Sensitivity (%)^a^	Sensitivity (%)^b^
Submicroscopic	31	20	11	n/a	65
< 1000	7	6	1	86	86
1000–10,000	13	11	2	85	85
> 10,000–< 50,000	11	11	0	100	100
50,000–< 100,000	9	9	0	100	100
> 100,000	7	7	0	100	100

*TP* true positive; *FN* false-negative

^a^Sensitivity of nPCR-saliva with TFM as reference standard

^b^Sensitivity of nPCR-saliva with nPCR-blood as reference standard

**Table 5:** Performance of nPCR-saliva across different age group

Test characteristics	Age group (years) (n)			
	2–10 (60)	11–20 (47)	21–39 (93)	> 40 (22)
Median parasitaemia/µl of blood	71,907	23,257	26,760	13,600
Sensitivity (%)	94	94	93	100
Specificity (%)	88	97	95	100
PPV (%)	76	89	78	100
NPV (%)	97	99	99	100

**Fig. 2:**
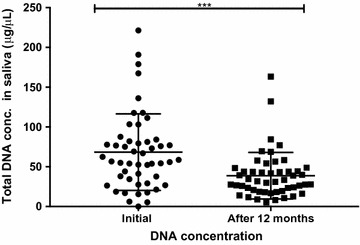
Comparison of total DNA concentration in *Plasmodium falciparum* positive saliva samples. DNA was extracted from 200 µl of saliva when they were initially collected and quantified. The saliva samples were left at room temperature for 1 year and DNA was re-extracted and quantified. ***P = 0.0002

To determine the stability of total DNA and *Pf* DNA in saliva samples at ambient temperature, samples were stored at room temperature for 12–13 months. Then, total DNA was extracted, quantified and nPCR-saliva repeated using the same reaction conditions as before. There was a significant decrease (P < 0.0001) in the total DNA concentration after 12–13 months (mean 38.7 ng/µl) as compared to the total DNA concentration when the samples were collected (mean 68.3 ng/µl) (Fig. 2). However, 80% of the saliva samples that were positive for *Pf* DNA at the time of collection remained positive 12–13 months later even though the samples were stored at room temperature (Table 6). The initial mean parasitaemia of saliva samples that remained positive after 12–13 months at room temperature was 50,221 parasites/ul of blood while it was 13,540 parasites/µl of blood in saliva samples that were initially positive but became negative 12–13 months later. This difference was statistically significant (P = 0.0065).

**Table 6:** Relationship between parasitaemia and nPCR-saliva after one year

Parasitaemia at enrolment (n)	Samples that remained positive after 1 year n (%)	Samples initially positive but negative after 1 year n (%)
Sub microscopic (14)	7 (50)	7 (50)
< 1000 (5)	5 (100)	0
1000–10,000 (11)	9 (82)	2 (18)
> 10,000–< 50,000 (11)	11 (100)	0
50,000–< 100,000 (7)	7 (100)	0
> 100,000 (6)	5 (83)	1 (17)

Originally, 53 samples were nPCR-saliva-positive. A year later, the samples were re-screened and n = 43 remained positive, but 10 were negative

## Discussion

Saliva is increasingly being recognized as a potential non-invasive alternative to blood for malaria diagnosis. Sample handling methods that do not compromise the quality of samples and do not require cold chains are ideal for field settings in low and middle-income countries. This study was conducted to assess the diagnostic performance of nPCR for detection of *P. falciparum* DNA in saliva collected into OMNIgene^®^•ORAL (OM-501) kit and stored at room temperature. Nested PCR-saliva was compared with two well-established blood-based methods for malaria diagnosis, i.e., TFM and nPCR-blood. The prevalence of malaria in the study population detected by nPCR-blood (35%) was higher than prevalence found by TFM (24%), agreeing with well-known increased sensitivity of nPCR-blood for diagnosis of malaria [4–6, 13–16], and confirming the existence of submicroscopic infections in the population. The number of malaria-positive individuals detected by nPCR-saliva (28%) was greater than that detected by TFM (24%), but lower than that detected by nPCR-blood (35%). Interestingly, nPCR-saliva detected 13 more malaria infections than TFM, which is currently the gold standard for malaria diagnosis.

More detailed parameters of diagnostic performance were used to compare nPCR-saliva with TFM and nPCR-blood. When TFM was used as gold standard, nPCR-saliva recorded a sensitivity of 95%, missing only three TFM-positive cases. A closer analysis of these three cases revealed that their parasitaemia was among the lowest in the study (i.e., 80, 1400 and 1800 parasites/µl of blood). Even though the mechanism of transfer of parasite DNA from blood to saliva is still unknown, it is plausible that higher amounts of parasite DNA are found in saliva when blood parasite densities are higher. Indeed, the sensitivity of nPCR-saliva decreased to below 100% only when parasitaemia ranged from 1000 to 10,000/µl (Table 4). The sensitivity of the assay used in this study (85%) is similar to the sensitivity reported previously of 85.2% by Buppan et al. [30] at parasitaemia greater than 5000/µl. Buppan and collaborators stored saliva samples at 4 °C while saliva samples in this study were left at room temperature in the OMNIgene^®^•ORAL (OM-501) kit. Taken together, the results show that the saliva collection kit successfully preserved parasite DNA in saliva at room temperature and detection of *P. falciparum* DNA in saliva except at the lowest parasitaemia suggests a passive mechanism of transport of *P. falciparum* DNA along a concentration gradient from blood to saliva.

When nPCR-blood was used as gold standard, nPCR-saliva recorded sensitivity and specificity of 82 and 99%, respectively, again showing that nPCR-saliva is potentially more sensitive than TFM for malaria diagnosis. However, a few samples showed a lack of concordance between nPCR-saliva and nPCR-blood. In the first case, two samples were positive for *P. falciparum* DNA in saliva but negative for DNA in blood. It is known that malaria parasite density fluctuates over a 48-hour period in blood and is not always detectable due to sequestration of infected erythrocytes and the immune response of the infected individual. The two samples may have been collected when parasitaemia was low or undetectable by TFM. Moreover, *P. falciparum*-infected red blood cells in blood circulate through the spleen where they are removed by splenic macrophages leading to clearance of parasites and their products. Saliva contains fewer macrophages than the spleen, and there is no evidence that infected red cells are present in saliva. Thus, *P. falciparum* DNA in blood may be cleared faster than from saliva. Because follow-up samples were not collected from study participants, it was not possible to determine how long parasite DNA in saliva circulates/persists after parasite clearance from the blood. In the second case, there were 14 samples (11 with undetectable parasites by TFM) for which DNA was detected in blood by PCR, but not in saliva. Some reasons can be evoked. Firstly, low parasitaemia as mentioned above may be associated with little or no DNA present in saliva. Secondly, the infections may have been newly acquired such that blood containing infected RBC or parasite DNA may not have circulated long enough through the salivary glands to infiltration of DNA into saliva. Thirdly, parasite sequestration into the deep tissue via adhesion of *Pf*EMP1 to CD36 can reduce the parasite load that transits through to the salivary gland blood vessels. Also, it is possible that infected erythrocytes sequester in the salivary glands through adhesion to CD36 on the salivary glands blood vessels.

It is important to note that although saliva samples were stored at room temperature, the nPCR-saliva performance in this study was slightly better than nPCR-saliva performance in other studies using saliva stored at 4 °C or − 20 °C [34, 38]. This suggests that saliva samples collected in the OMNIgene^®^•ORAL (OM-501) kit used in this study and stored at ambient temperature are as reliable as frozen samples.

For the 14 cases for which DNA was positive in blood but negative in saliva, none of the individuals were > 40 years (5 were 2–10 years, 3 were 11–20 years, and six were 21–39 years old). This prompted the investigation of any age-related differences in the sensitivity of nPCR-saliva. Moreover, most previous studies have only enrolled individuals aged 10 years and above. Age-stratifications showed that the sensitivity of nPCR-saliva ranged from 94 to 100% (Table 5) confirming that nPCR-saliva can be used in all age groups.

Finally, 1 year after sample collection, *P. falciparum* DNA could still be detected in 80% of saliva samples that had been stored at room temperature. Even though there was a significant decrease in the total DNA concentration in the saliva samples, only ten samples that were initially positive by nPCR-saliva were negative 1 year later. Of the ten samples, seven had sub-microscopic parasitaemia when collected. Thus, parasite DNA in saliva samples with low or submicroscopic parasitaemia may undergo some degradation. However, low parasitaemia did not seem to be the critical factor because some the samples with submicroscopic parasitaemia remained positive after 1 year. Taken together, the results show that the saliva collection kit used for this study can preserve *P. falciparum* DNA at room temperature for more than 1 year. Therefore, the use of DNA stabilization for the collection of saliva samples may eliminate the need for cold chain or dry ice in transporting samples from the field or shipping samples abroad thereby, reducing cost. The technology described in this study for diagnosis of malaria in resource-limited countries adds on to the armamentarium needed for elimination of malaria.

## Conclusions

The results of this study support the conclusion that nPCR-saliva is more sensitive than TFM for malaria diagnosis, at least in patients with fever. Furthermore, the results demonstrate that nPCR on saliva collected from individuals of different ages and stored in the OMNIgene^®^•ORAL (OM-501) kit at room temperature are equally, or more accurate than results previously-reported for nPCR on saliva stored at 4 °C or − 20 °C. Even after 1 year, *P. falciparum* DNA could still be detected in saliva samples stored at ambient temperature. With the capacity to store *Plasmodium* DNA at ambient temperature and the non-invasive sample collection procedure, OMNIgene^®^•ORAL, saliva-based nPCR presents a new diagnostic tool for clinical trials and epidemiological surveillance studies where submicroscopic infections need to be detected.

## Abbreviations

HRP-2: histidine-rich protein-2
nPCR: nested PCR
NLR: negative likelihood ratio
NPV: negative predictive value
PCR: polymerase chain reaction
PLR: positive likelihood ratio
PPV: positive predictive value
RDT: rapid diagnostic test
SSA: Sub-Saharan Africa
TFM: thick film microscopy
